# Disseminated Skeletal Angiomatosis Initially Misdiagnosed As Metastatic Tumor: A Case Report

**Published:** 2017-01-02

**Authors:** Fariba Binesh, Kazem Aghili, Marjan Hakiminia, Mohammad Reza Vahidfar, Roghayeh Masumi

**Affiliations:** 1 *Dept. of Pathology, Shahid Sadoughi University of Medical Sciences, Yazd, Iran*; 2 *Dept. of Radiology, Shahid Sadoughi University of Medical Sciences, Yazd, Iran*; 3 *Dept. of Internal medicine, Shahid Sadoughi University of Medical Sciences, Yazd, Iran*; 4 *Blood and Cancer Research Center, Shahid Sadoughi University of Medical Sciences, Yazd, Iran*

**Keywords:** Angiomatosis, Disseminated, Skeletal, Misdiagnosis

## Abstract

Disseminated angiomatosis, also referred to as cystic angiomatosis, is a generalized disease that involves bones and soft tissue. It is characterized by multifocal hemangiomatous lesions of the bones with possible visceral organ involvement. The clinical manifestations differ according to the site and the extension of disorder. Here we describe a case of generalized angiomatosis occurring in a 35-yr-old Iranian woman, initially misdiagnosed as osseous metastasis, who presented at Shahid Sadoughi Hospital, Yazd, Iran in February 2014. Although the clinical, radiological and pathological features are diagnostic, the process is often mistaken with other lytic lesions of the bones, especially malignant tumors. The case is being reported owing to its scarcity as per the literature published globally.

## Introduction

Angiomatosis is an extremely rare entity characterized by thin-walled blood vessel proliferation with bone destruction. It is determined as replacement of bone or soft tissue by hemangiomatous or lymph angiomatous tissue (1). The term cystic angiomatosis is applied to very rare conditions of generalized multifocal hemangiomas in the skeleton. The bone involvement may be accompanied by soft tissue hemangiomas. The sites of extraskeletal involvement include lung, liver, and especially spleen. Most patients are diagnosed in the first three decades of life. The disease can be asymptomatic and is detected incidentally in radiographs obtained for other reasons (2). 

Although skeletal angiomatosis, as a rule, has a benign course, the differential diagnosis includes more disorders aggressive, including malignant lesions. Precise imaging studies, including MRI, can be performed to better recognize a lesion as angiomatosis (3). Owing to its rarity, angiomatosis should be kept as one of the rare differential diagnosis in a patient presenting with multiple lytic lesions of bone. 

Here we report a rare case of diffuse skeletal angiomatosis occurring in a 35-year-old female initially misdiagnosed as myelofibrosis probably secondary to osseous metastasis.

## Case report

**Fig. 1:**
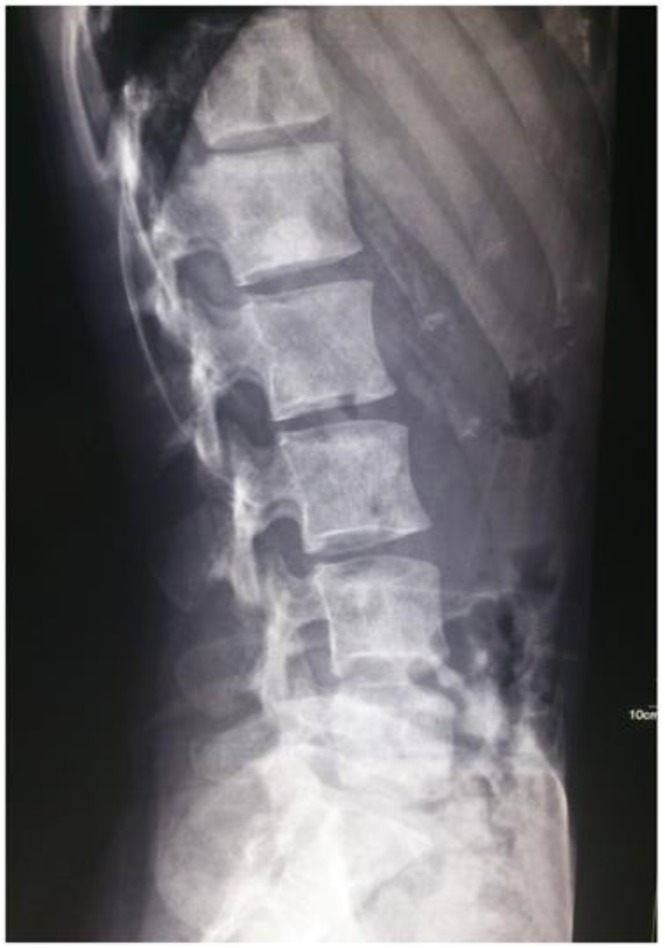
X=ray shows multiple well defined sclerotic lesions, without bone destruction or lytic component

**Fig. 2 (A &B).**
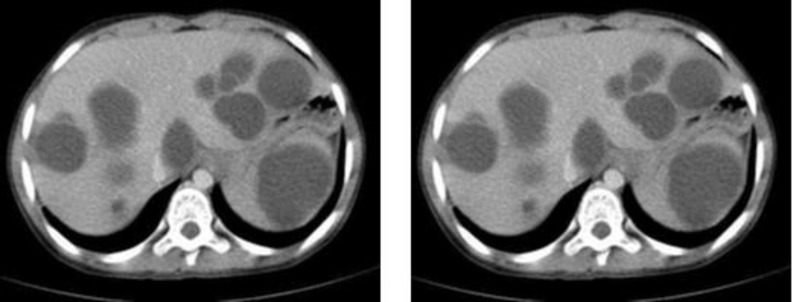
Abdominal CT scan (portal phase) with contrast reveals multiple well-defined low-attenuation mass lesions in spleen and liver without marked enhancement

**Fig 3 (A & B).**
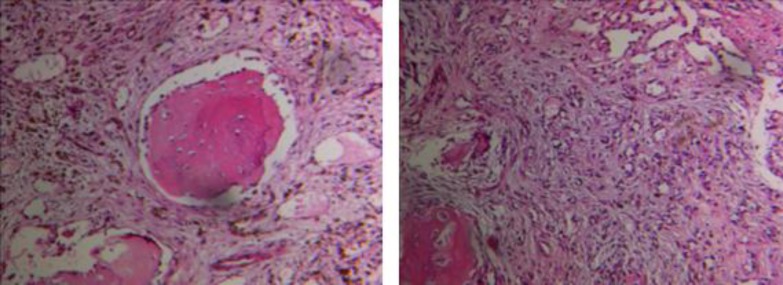
Shows thin-walled vessels within the marrow spaces of cancellous bone with associated reactive fibrosis (H&E stain X40).

**Fig. 4:**
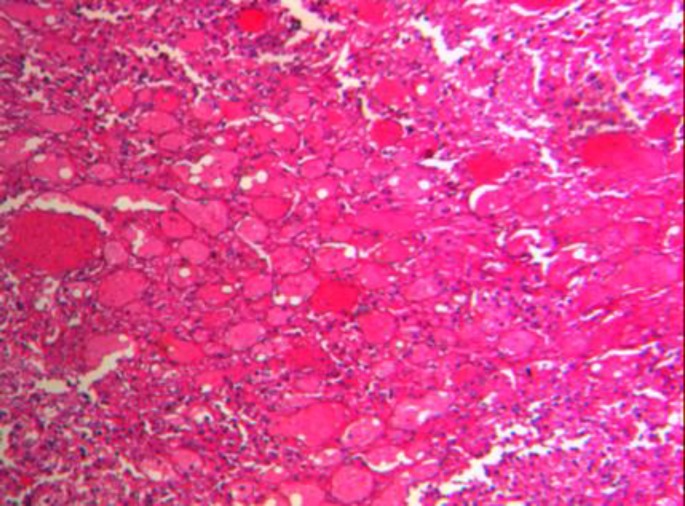
Reveals spleen tissue with tumor composed of larger vessels with cystically dilated lumina and thin walls (H&E stain X 40

A 35-year-old woman presented to an outside medical clinic in Yazd, Iran, in January 2012 with low back pain. Her pain was started since several years ago. Physical examination had shown moderate splenomegaly without any evidence of lymphadenopathy or hepatomegaly. Routine blood test results were normal, except an elevated level of alkaline phosphatase (ALP=350U/ l, normal: 20–125 U/l). Radiographs had demonstrated numerous lesions throughout the lumbar vertebrae (Fig. 1) and skull (not shown here), suggestive of osseous metastases. A bone scan had shown avid radioisotope uptake in the mentioned areas. Abdominal CT-scan with contrast had shown multiple well-defined low-attenuation mass lesions in spleen and liver without marked enhancement (Fig. 2A,B). Biopsy of the posterior iliac crest was performed at that time reported as myelofibrosis probably secondary to osseous metastasis. Subsequently, the patient had undergone splenectomy in a private hospital. The pathology report of splenectomy material was malignant vascular tumor. As a result, the patient had received 6 cycles of chemotherapy. In order to severe pain in lumbar area and lower extremities that were unresponsive to chemotherapy, the patient referred to our center in February 2014. All the pathology slides were subsequently reviewed at our institution in conjunction with the radiographic studies. The pathologic features of the posterior iliac crest biopsy showed thin-walled vessels within the marrow spaces of cancellous bone with associated reactive fibrosis (Fig.3A,B). These vascular spaces were dilated, filled with blood, and lined by bland endothelial cells without nuclear pleomorphism or mitotic activity to suggest a malignant vascular neoplasm. The findings were felt to be, instead, characteristic of angiomatosis of bone. The slides related to splenectomy material revealed spleen tissue with tumor composed of larger vessels with cystically dilated lumina and thin walls. Foci of old hemorrhage, fibrosis, and calcium deposition were also present (Fig. 4). There was no evidence of malignancy. The final diagnosis was generalized angiomatosis.

## Discussion

Skeletal angiomatosis is defined as proliferation of benign looking vascular channels that occurs in bone and sometimes extra skeletal tissues. In disseminated angiomatosis or so-called cysic angiomatosis, there is hamartomatous proliferation of immature vessels (4) that mainly involves the trunk bones and sporadically calvarium (2). However, Ratna Kumari Beeram et al. described a 15- year-old boy with angiomatosis of craniofacial that was progressive. In addition the patient suffered from severe anemia due to accompanied splenomegaly (5). 

A case of angiomatosis of maxilla and mandible was described in a young male (6). A patient with pelvic angiomatosis was reported as a very rare cause of recurrent obstructed labor (7). An unusual combination triad of congenital malformations including skeletal cystic angiomatosis, A-V malformations, and Arnold-Chiari malformation type I was reported (8) . The age of onset has been reported from 1 month to 75 yr with a peak in the second and third decades (9). For example, one patient was reported at 71 yr of age (10). Three patients were reported over the age of 50 (11). The patient in this case report was 35 yr old, however, her symptoms had begun from several years ago.

 This condition has two etiologies, a congenital and an acquired.The pathogenesis of angiomatosis remains obscure, but now, it is believed to result from vascular malformations of congenital origin (12). Osseous involvement occurs most commonly in the spine and calvaria and the disease tends to waive the acral appendicular bones. The thoracic spine is the most common location for vertebral involvement (1). It was true in our case. Angiomatosis can involve predominantly osseous or soft tissues**.** Although isolated skeletal angiomatosis could occur (13), concomitant disease of the non-osseous tissues exists in 60–70% of cases (14), usually in the spleen (12). Our patient had skeletal and extra skeletal involvement.

 The process may be asymptomatic or progressing rapidly till the bone is replaced by fibrous connective tissue, surrounding a cavity composed of thin-walled capillaries. The current case complained of pain. There is no particular sex or race predilection and no associated endocrine, metabolic or immunological disorder was known (15). However, the first case of the triad of cystic angiomatosis of bone, angiomatosis of the viscera and paraproteinemia was indicated (9). In addition, three cases of angiomatosis in patients above were diagnosed the age of 60 yr (11). In one of these cases, malignant lymphoma developed. We did not find any associated disease in our patient. Radiographic findings in cystic angiomatosis include lytic bone lesions with some degree of reactive woven bone formation.

The lesions are typically radiolucent but occasionally sclerotic. It was true in our case. In rare instances, the disorder is manifested on radiographs as disseminated osteoblastic lesions that mimic osteoblastic metastases. A case of skeletal angiomatosis occurring in a 35-year-old Caucasian woman was described, and documented the temporal progression of the disease over a 15-year period (16). In other words, evaluation of serial radiographs in this disorder has shown that increasing osteosclerosis can be an age-related phenomenon in angiomatous foci that are originally lytic.

 The sclerotic lesions occur most often in older patients. CT has traditionally been considered the diagnostic technique of choice and demonstrates multiple low-density cysts with or without calcifications (17). X-Ray of the spine in the present case showed sclerotic lesions. Differential diagnoses are lymph angiomatosis, Gorham's disease, multiple myelomas, skeletal metastases, eosinophilic granuloma, fibrous dysplasia, and enchondroma (8). In the current case, imaging modalities were more in favor of angiomatosis. Although imaging studies propose significant profit in making the diagnosis, histological examination is finally needed to confirm the diagnosis (13). In other words, diagnosis is difficult and requests a biopsy.

 Careful attention to pathologic changes is imperative in instances of widespread angiomatosis, because the radiologic appearance may closely resemble metastatic tumors (16). The skeletal lesions in generalized angiomatosis are usually stable or may even regress. The lesions spontaneously heal that this process is accompanied by sclerosis. Visceral lesions cause fatal complications (2).

 Treatment of the skeletal lesion has been conservative; however, very good therapeutic effect for combined interferon alpha, thalidomide, and zoledronate in the case of multiple angiomatoses has been reported (18). Our patient received conservative therapy. A patient affected by skeletal angiomatosis of bone treated with surgery and then with amino bisphosphonates has been described. This therapy resulted in an immediate effectiveness in reducing bone pain and stable radiological findings during clinical follow-up (19). Angiomatosis shows no tendency toward malignant transformation (20) but malignant de-differentiation may occur following radiation therapy (21). 

## Conclusion

Angiomatosis is a rare congenital disease involving the vascular and lymphatic systems**.** Its differential diagnosis includes more aggressive disease processes, including malignant lesions**.** This case is presented for its rarity and differential diagnosis challenge.

## Conflict of Interests

The authors declare that there is no Conflict of Interests. 
